# A Nanoclay-Enhanced Hydrogel for Self-Adhesive Wearable Electrophysiology Electrodes with High Sensitivity and Stability

**DOI:** 10.3390/gels9040323

**Published:** 2023-04-11

**Authors:** Fushuai Wang, Lang Yang, Ye Sun, Yiming Cai, Xin Xu, Zhenzhong Liu, Qijie Liu, Hongliang Zhao, Chunxin Ma, Jun Liu

**Affiliations:** 1 Key Laboratory for Biomedical Engineering of Education Ministry, Department of Biomedical Engineering, Zhejiang University, Hangzhou 310027, China; 2 State Key Laboratory of Marine Resource Utilization in South China Sea, Hainan University, Haikou 570228, China; 3 Taizhou Key Laboratory of Medical Devices and Advanced Materials, Research Institute of Zhejiang University-Taizhou, Taizhou 318000, China; 4 Key Laboratory of Quality Safe Evaluation and Research of Degradable Material for State Market Regulation, Products Quality Supervision and Testing Institute of Hainan Province, Haikou 570203, China

**Keywords:** electrophysiology signals, hydrogel electrodes, nanoclay, self-adhesion, wearable sensors

## Abstract

Hydrogel-based wet electrodes are the most important biosensors for electromyography (EMG), electrocardiogram (ECG), and electroencephalography (EEG); but, are limited by poor strength and weak adhesion. Herein, a new nanoclay-enhanced hydrogel (NEH) has been reported, which can be fabricated simply by dispersing nanoclay sheets (Laponite XLS) into the precursor solution (containing acrylamide, N, N′-Methylenebisacrylamide, ammonium persulfate, sodium chloride, glycerin) and then thermo-polymerizing at 40 °C for 2 h. This NEH, with a double-crosslinked network, has nanoclay-enhanced strength and self-adhesion for wet electrodes with excellent long-term stability of electrophysiology signals. First of all, among existing hydrogels for biological electrodes, this NEH has outstanding mechanical performance (93 kPa of tensile strength and 1326% of breaking elongation) and adhesion (14 kPa of adhesive force), owing to the double-crosslinked network of the NEH and the composited nanoclay, respectively. Furthermore, this NEH can still maintain a good water-retaining property (it can remain at 65.4% of its weight after 24 h at 40 °C and 10% humidity) for excellent long-term stability of signals, on account of the glycerin in the NEH. In the stability test of skin–electrode impedance at the forearm, the impedance of the NEH electrode can be stably kept at about 100 kΩ for more than 6 h. As a result, this hydrogel-based electrode can be applied for a wearable self-adhesive monitor to highly sensitively and stably acquire EEG/ECG electrophysiology signals of the human body over a relatively long time. This work provides a promising wearable self-adhesive hydrogel-based electrode for electrophysiology sensing; which, will also inspire the development of new strategies to improve electrophysiological sensors.

## 1. Introduction

Stably and highly sensitively acquiring bioelectrical signals, including electromyography (EMG), electrocardiogram (ECG), and electroencephalography (EEG), is mostly important for detecting and treating the human body [1,2,3,4]. As the key component, the electrode that non-invasively obtains signals has mostly two types: a wet electrode and a dry electrode [5,6]. The wet electrode, as the gold standard, is composed of the Ag/AgCl electrode and conductive paste or hydrogel which can contact and infiltrate the surface of skin [7,8]. Owing to reduced skin–electrode impedance, the wet electrode can obtain high-quality and stable signals. Furthermore, the wet electrode commonly has stable electrolytic half-cell potential at the contact interface between the Ag/AgCl electrode and conductive paste [5]. The Ag/AgCl electrode has excellent anti-interference ability owing to its quickly stabilized electrolytic half-cell potential. One type of wet electrode is the commercial electrode patch (CEP), which is composed of the Ag/AgCl electrode and hydrogel. Another type is the disc electrode, which is composed of a disc-shaped Ag/AgCl electrode. The two types of wet electrodes are widely used in clinical/laboratory applications for acquiring high-quality signals. However, the signals of the wet electrode worsen with the water evaporation of the paste or hydrogel [9]. Furthermore, using the disc electrode is very inconvenient, commonly requiring assistance and leading to difficulty in cleaning the skin after use. The dry electrode is usually composed of conductive materials, such as metals and conductive polymers, which are convenient to use without the conductive paste and suitable for long-term monitoring without the water evaporation problem [10,11]. However, the dry electrode has relatively higher skin–electrode impedance and lower signal quality [9,12]. In general, to conveniently acquire high-quality stable signals, electrodes urgently need to be greatly improved and developed.

Among various dry electrode materials, hydrogel has unique advantages owing to its biological, tissue-like soft and wet structure, which is outstandingly biocompatible and skin compliant for use on human skin [13,14,15]. As is known to all, various hydrogel-based dry electrodes have been successfully developed for acquiring EMG, ECG, and EEG biosignals through adding electron-conductive materials into hydrogels [16,17,18,19]. For instance, Hsieh [20] designed a polyethylene glycol (PEG) hydrogel electrode with low skin–electrode impedance and long-term electrical stability. Sun [21] reported a copoly(acrylate-acrylic acid) hydrogel electrode with excellent water-resistant, underwater adhesion and swelling resistance for application to human skin in the aquatic environment. Luo [22] presented an MXene-poly(acrylic acid) composite hydrogel electrode which can provide excellent skin compliance. However, these hydrogel-based dry electrodes commonly have relatively unstable and low-sensitive biosignals, due to their electron conduction and subsequently high skin–electrode impedance. Compared with the hydrogel-based dry electrodes above, hydrogel-based wet electrodes based on ionic conductivity can have relatively high sensitivity and biosignal stability; which, have attracted more and more attention. Recently, a few of hydrogels for commercial wet electrodes have been used in clinical applications; however, they generally have low strength and weak self-adhesion. Therefore, low strength of these hydrogels makes it difficult for these wet electrodes to be firmly fixed on the skin and to efficiently resist disturbances such as motion and vibration. Additionally, the weak self-adhesion of these hydrogels leads to the addition of pressure-sensitive adhesives on these electrodes which have poor biocompatibility and can cause inflammation or allergy of human skin [23]. Most recently, Shen [24] developed a poly(N-acryloyl glycinamide) hydrogel-based wet Ag/AgCl electrode with high strength but without self-adhesion. Nowadays, it is still a huge challenge to explore new hydrogel for high-sensitivity wet bioelectrodes which can integrate high strength, long-term stability, and self-adhesion together.

**Scheme 1:**
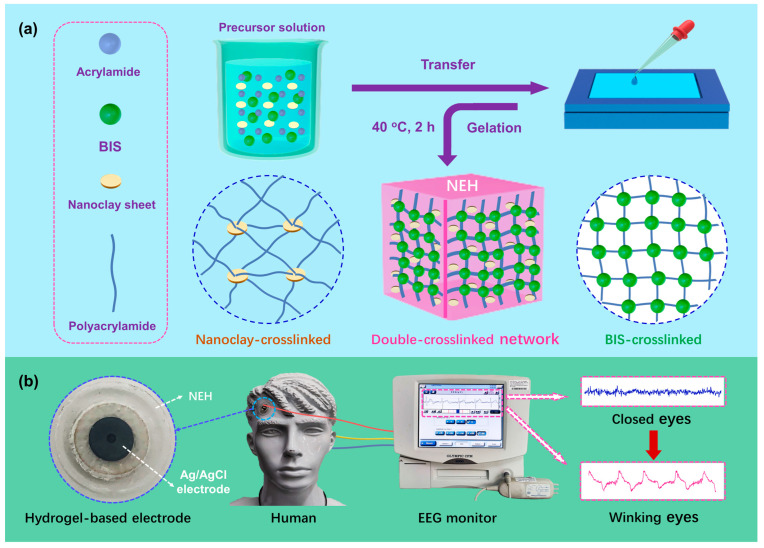
(**a**) Schematic illustration for the double-crosslinked network of Nanoclay-Enhanced Hydrogel and the fabricating process. (**b**) Application of the NEH self-adhesion electrode for electrophysiology signal acquisition.

Herein, a new hydrogel-based wet electrode has been reported which has nanoclay-enhanced strength and self-adhesion to achieve electrophysiologic signals that are highly sensitive and stable in the long term (Scheme 1). This nanoclay-enhanced hydrogel (NEH) was fabricated simply by dispersing nanoclay sheets (Laponite XLS) into the precursor solution (containing acrylamide, N,N′-Methylenebisacrylamide, ammonium persulfate, sodium chloride, glycerin) and then thermo-polymerizing at 40 °C for 2 h (Scheme 1a). Nanoclay can enhance the self-adhesive and mechanical properties of the hydrogel, forming a composite network in the hydrogel; which, can firmly adhere onto human skin to achieve a complete signal-acquiring pathway and subsequently low skin–electrode impedance to efficiently resist motion disturbances. Furthermore, glycerin in hydrogel can decrease water loss rate to enhance the ionic–conductive (3% NaCl) stability for acquiring signals. Therefore, this NEH hydrogel-based Ag/AgCl wet electrode, with a stainless steel buckle and backing material, can be an excellent wearable self-adhesive monitor to highly sensitively and stably acquire EEG/ECG electrophysiology signals for a relatively long time without obvious disruption/discomfort (Scheme 1b).

## 2. Results and Discussion

### 2.1. Basic Characterization of NEH

**Figure 1:**
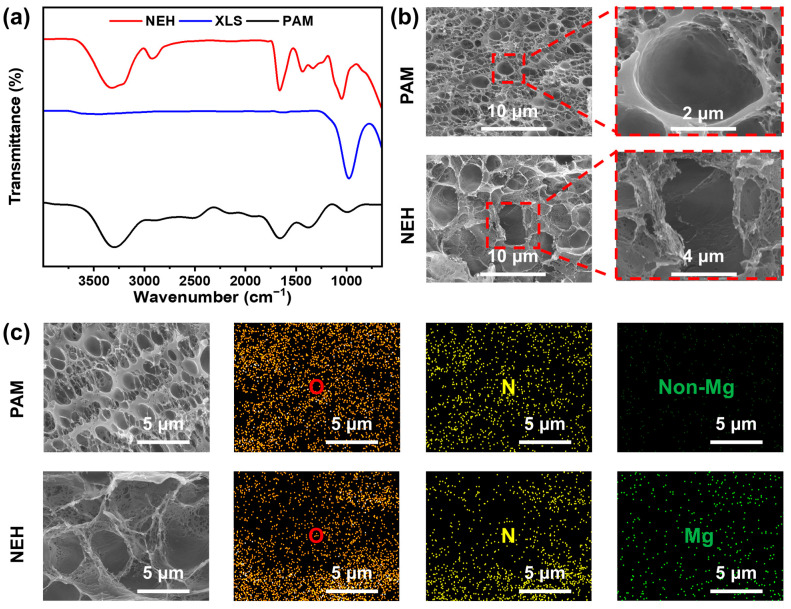
(**a**) FT-IR spectra of NEH, XLS, and PAM. (**b**) SEM and (**c**) EDS mapping of PAM and NEH.

The basic properties of the NEH were characterized and researched by Fourier Transform Infrared Spectrometer (FT-IR spectra), Scanning Electron Microscope (SEM), and Energy Dispersive Spectroscopy (EDS) mapping (Figure 1). In Figure 1a, the FT-IR spectra of polyacrylamide (PAM), nanoclay (XLS), and NEH were characterized. The characteristic absorption peaks of PAM at 3328 cm^−1^, 1669 cm^−1^, and 996 cm^−1^ were free -NH_2_, a carbonyl, and C=O stretching vibration, respectively. The characteristic peak of XLS at 1631 cm^−1^ was the bending vibration of interlayer water; while, the strong absorption band at 966 cm^−1^ was Si-O-Si stretching vibration. Notably, both the characteristic peaks of PAM and XLS appeared in the spectrum of NEH, which confirmed that XLS has been fixed in NEH. In order to investigate the dispersion state of nanoclay within NEH, we further used SEM and EDS mapping to conduct morphological analysis. As shown in Figure 1b, it can be seen that the distribution of pores on the surface of the hydrogel without nanoclay was more uniform than hydrogels with nanoclay. From the enlarged SEM (red dotted line), the size of the pores was regular and the wall was smooth for hydrogel without nanoclay; while, the composite hydrogels with nanoclay were irregular and rough. There were small holes in the original hole on the surface of NEH, forming a complex three-dimensional network. EDS mapping was further used to conduct element analysis of hydrogels (Figure 1c). Both hydrogels contained nitrogen and oxygen elements. However, only the hydrogel with nanoclay contained Mg element because the nanoclay sheet (Laponite XLS) is mainly composed of phyllosilicates which contained a large amount of Mg element [25]. The results of EDS mapping further proved that nanoclay had filled in the polyacrylamide hydrogel. Besides, we conducted the experiment to prove that nanoclay in NEH can be another crosslinker except for BIS. As shown in Figure S5, when no BIS was added in the precursor solution, the nanoclay can still activate the gelation by itself. Therefore, NEH was the composite hydrogel crosslinked by BIS and nanoclay. This result was consistent with previous studies [26,27].

### 2.2. Mechanical Properties of NEH

**Figure 2:**
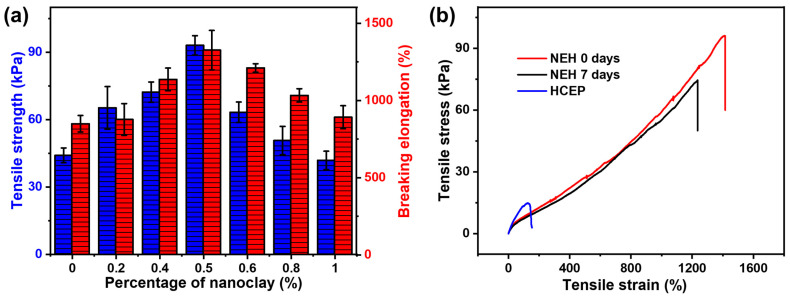
(**a**) Tensile strength and breaking elongation results of NEH with different contents of nanoclay. (**b**) The tensile stress–strain curves of NEH at 0 days and 7 days, compared with HCEP.

The application of traditional hydrogel is severely limited by its poor mechanical properties [28]. In order to study the effect of nanoclay content on the mechanical properties of the NEH, we prepared NEH with different nanoclay concentrations and analyzed their mechanical performance (Figure 2). As shown in Figure 2a, it can be seen that the change of nanoclay had a significant effect on the breaking elongation and tensile strength of the NEH. As the content of nanoclay increased from 0% to 0.5%, the breaking elongation increased from 850% to 1326% and the tensile strength increased from 44 kPa to 93 kPa. However, with further increase of nanoclay content, the values of breaking elongation and tensile strength decreased significantly. To study the reason for this change of tensile strength with the increase of nanoclay content, we conducted an experiment to observe the stability of nanoclay in precursor solution: the optical photos indicated that excessive nanoclay (mass percentage of nanoclay > 0.5%) could make the precursor solution turbid (Figure S6); which, indicated the aggregation of nanoclay sheets. Furthermore, the test of UV-Vis spectrophotometer showed the light transmittance (400–800 nm) sharply decreased when nanoclay content increased from 0.5 to 0.8%; which, also manifested the aggregation of nanoclay sheets (Figure S7). Therefore, the change of the tensile strength and breaking elongation with the increase of nanoclay contents can be explained: when the nanoclay content was less than 0.5%, it can disperse with a single nanosheet state in the precursor solution; which, can highly enhance the tensile strength and the breaking elongation of the hydrogel with the increase of the nanoclay content. When the nanoclay content was greater than 0.5%, the nanoclay sheets can aggregate and cannot be dispersed well in the precursor solution; which, may decrease the tensile strength and breaking elongation. In addition, similar to the BIS crosslinker, the nanoclay sheet can also be a crosslinker for the acrylamide monomer [26,29,30]; which, indicates this NEH can has a double-crosslinked network with high mechanical strength. Therefore, we preliminary chose 0.5% of nanoclay content to fabricate the NEH hydrogel. Simultaneously we tested the mechanical properties of NEH-0.5% (which refers to the nanoclay concentration in the NEH being 0.5%) after 7 days. The results showed that although the breaking elongation and tensile strength at breaking of NEH-0.5% after 7 days decreased, it was still better than HCEP, indicating that NEH-0.5% had good toughness and stability (Figure 2b).

### 2.3. Adhesive Performance of NEH

**Figure 3:**
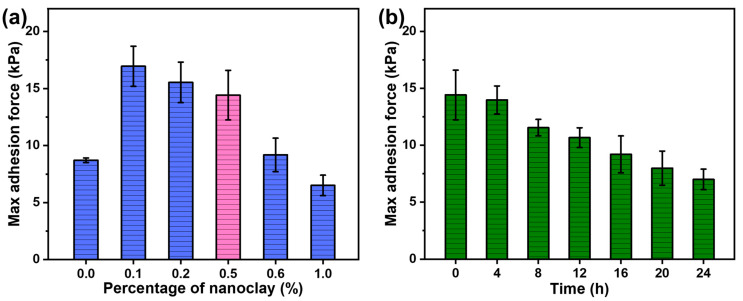
(**a**) Adhesive property for NEH with different percentage contents of nanoclay (The pink column is the selected nanoclay content for the following study). (**b**) Comparison of adhesive property on the glass substrate for 24 h at 25 °C, 65% humidity.

In order to investigate the adhesive performance of NEH, we studied the adhesive performance on different substrates, e.g., glass, silicone, stainless steel, pigskin, PVC, PP, PC, and PET (Figure 3). We selected glass substrate to test the adhesive performance (17 kPa) because it can obtain stable data for the multi-repeated experiment, compared with results on the other substrates (8.5 kPa on silicone, 12 kPa on stainless steel, 6 kPa on pigskin, 13 kPa on PVC, 11 kPa on PP, 12 kPa on PC, 11 kPa on PET) (Figure S2). As shown in Figure 3a, the adhesive force of hydrogel without nanoclay was 9 kPa; with the increase of nanoclay content (from 0% to 1.0%), the adhesion force first increased (0.1%) and then decreased (>0.1%) gradually. This interesting phenomenon can be explained: as a nanomaterial, added nanoclay sheets without reaction can enhance the adhesion of the hydrogel [29,30]. However, because the nanoclay sheet can also be a crosslinker for the acrylamide monomer, with its further increase, the crosslinked network can significantly become highly dense which can severely limit the movement of the polyacrylamide chain segments and subsequently decrease the adhesion of the hydrogel [29,30]. Therefore, the adhesion of this NEH had a maximum at 0.1% of nanoclay content. However, the NEH with 0.1% of nanoclay content had relatively low strength because of the low density of the crosslinked network; while, the NEH with 0.5% of nanoclay content had maximum mechanical strength and near maximum adhesion. Under comprehensive consideration, we finally selected the NEH with 0.5% of nanoclay content. Furthermore, the adhesion curves of the NEH-0.5% for 24 h indicated that the prepared NEH still had good adhesive property after 24 h at room temperature; which, was nearly the same as the fresh hydrogel without nanoclay (Figure 3b). Finally, we chose 0.5% as the final nanoclay concentration considering the mechanical and adhesive properties.

### 2.4. Water Retention Performance of NEH

**Figure 4:**
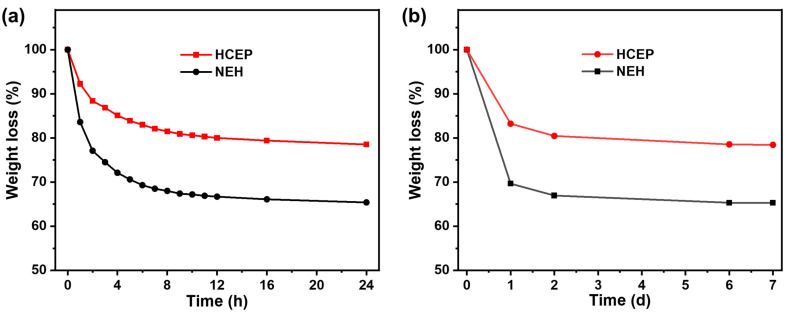
The weight loss curves of NEH and HCEP. (**a**) For 24 h, at 40 °C, 10% humidity, and (**b**) for 7 d at 20 °C, 20% humidity.

Water retention is greatly important for the stable adhesion at the skin–electrode interface; which, contributes to keeping the contact impedance and complete signal-acquiring path [17]. So, water retention performance is a precondition for the stability of hydrogel. We set two experiments to test the water retention performance of NEH compared with HCEP, which was tested and researched (Figure 4). As shown in Figure 4a, the weight loss rate of NEH was bigger than that of HCEP at 40 °C, 10% humidity, and 65.4% of NEH remained after 24 h. Besides, 65.3% of NEH and 78.5% of HCEP remained at 20 °C, 20% humidity for 7 days (Figure 4b). As we all know, when water evaporates, the hydrogel cannot maintain the tight adhesion; which, results in instability at the skin–electrode interface to influence signal quality and stability [22]. In our experiments, although the weight of NEH decreased more quickly than that of HCEP, the results also exhibited a certain water retention performance of NEH owing to the added glycerin.

### 2.5. Conductivity of NEH

The ionic conductivity of NEH was analyzed by electrochemical impedance spectroscopy (EIS). The results in Figure S4 showed that the conductivity of NEH with NaCl (1.3 S m^−1^) was much higher than that of NEH without NaCl (0.1 S m^−1^), indicating that the conduction mechanism of NEH mainly depends on the ionic conductivity of Na^+^, Cl^−^. Besides, the conductivity of NEH was tested at 25 °C, 65% humidity for 3 days. The conductivity of NEH was almost maintained at a stable level (0.6 S m^−1^) after decreasing within one day. NaCl can be completely ionized in water, but can only be ionized at a very low degree in glycerin; although, it can dissolve in glycerin [31]. The water was evaporated and the content of glycerin in the hydrogel increased, which can decrease the ionization degree of NaCl and tend to the reduction of hydrogel conductivity. In addition, although the water evaporation can increase the content of NaCl, which tends to increase hydrogel conductivity, perhaps this increase in conductivity cannot compensate for the reduction of conductivity. Therefore, with the evaporation of water, the conductivity of this NEH decreased to a certain extent.

### 2.6. Measurement of Impedance

**Figure 5:**
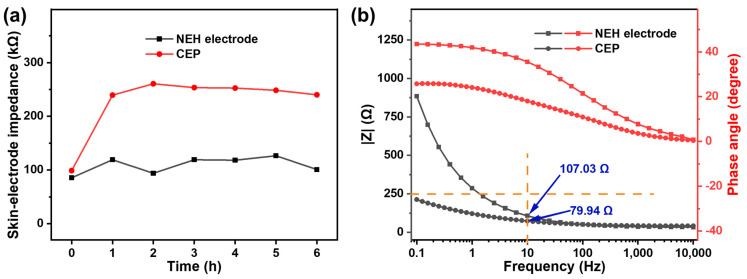
(**a**) Comparison of skin–electrode contact impedance at the forearm between the CEP and NEH electrode for 6 h. (**b**) Measurement for the electrode pair impedance of the NEH electrode and CEP.

The skin–electrode impedance affects the bioelectrical signal quality. The impedance test of NEH was divided into two parts: the skin–electrode impedance and the electrode pair impedance (Figure 5). During the skin–electrode impedance test, we used the NEH electrode as the working electrode and stuck it on the arm without removing it for 6 h, while two CEPs were used as the counter electrode and the GND electrode. The measured results at 10 Hz were shown in Figure 5a; the NEH electrode was more stable at nearly 100 kΩ. While tested in the same way, the CEP cannot maintain its initial low impedance (a bit higher than that of the NEH electrode). The impedance of the electrode itself refers to the impedance between two Ag/AgCl electrodes with hydrogel sandwiched in the middle. The impedance at 10 Hz of the NEH electrode pair and the CEP pair are 107.03 Ω and 79.94 Ω, both smaller than 250 Ω (Figure 5b). The results demonstrated the low impedance of the NEH electrode (at 10^0^–10^5^ Hz). In summary, NEH is a relatively ideal hydrogel for electrodes used in the long-term signal acquisition process.

### 2.7. EEG Recording by the NEH Self-Adhesive Electrodes

**Figure 6:**
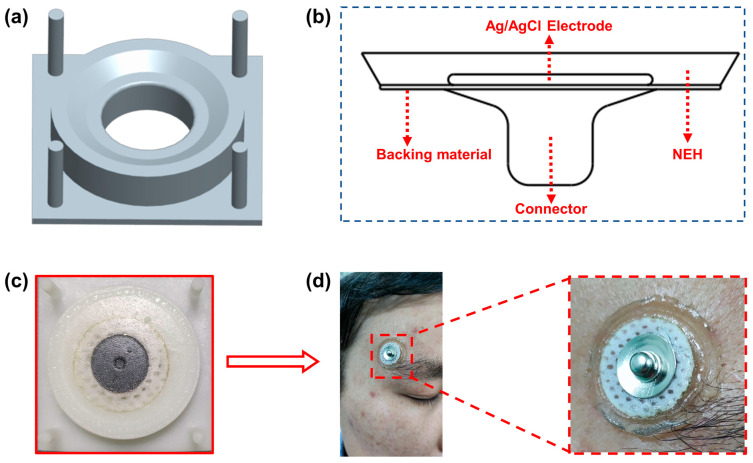
(**a**) Schematic illustration for self-adhesive electrode mold. (**b**) Schematic illustration of the structure of the NEH self-adhesive electrode. (**c**) Image of the NEH self-adhesive electrode manufacturing process. (**d**) Image of NEH self-adhesive electrode adhering to the skin of forehead.

Current CEPs have non-stretchable and poor-strength hydrogels [32]. Therefore, these hydrogels need to be combined with pressure-sensitive adhesives; which, will cause minor damage to the human body after long-term contact. As shown in Figure 6, we designed an NEH self-adhesive electrode with nanoclay-enhanced hydrogel composed of biocompatible materials [33]. The mold of the electrode was shown in Figure 6a, with four small cylinders distributed around the mold to support the sealed bag. The NEH self-adhesive electrode was composed of NEH, the Ag/AgCl electrode, stainless steel buckle (connector), and backing material (Figure 6b). The backing material included non-woven fabric and PET film. The non-woven fabric can enhance the bonding force between the backing material and NEH. In addition, the PET film was to prevent the leakage of precursor solution from the mold during the gelation process and reduce the evaporation area. The substrate was first placed in the mold consisting of the back material, the Ag/AgCl electrode, and the buckle. Then, we added the precursor solution into the mold, as shown in Figure 6c,d which shows the NEH self-adhesive electrode pasted to the skin and a magnified view of the NEH self-adhesive electrode (red dotted line).

**Figure 7:**
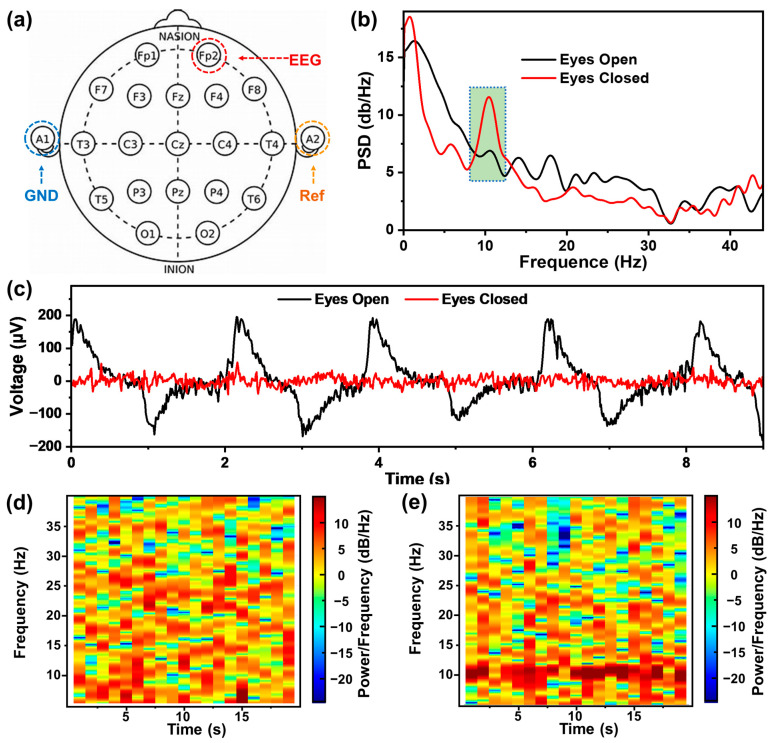
(**a**) Schematic illustration of electrode placement for the eye-open/eye-close EEG signal recording. (**b**) EEG power spectral density (PSD) of the eyes-open and eyes-closed periods. (The dashed box is to highlight the peak at 10 Hz.) (**c**) One channel EEG signal recorded during eyes blinking and rest. The time–frequency analysis of the signal from 0.5–45 Hz during (**d**) eyes open and (**e**) closed.

Compared with ECG and EMG, EEG signals are so weak (at μV level) that it is easy to be interfered with noise [34]. Thus, we conducted the experiment of collecting EEG to prove the feasibility of the NEH self-adhesive electrode (Figure 7). According to the 10–20 international lead standard [35], the NEH self-adhesive electrode was fixed to the Fp2 position as the EEG channel, and the Ref electrode and the GND electrode were located behind the left and right ears, respectively (Figure 7a). EEG signals in two states of eye open and eye closed were measured. Figure 7b shows the power spectra of signals in the two states. As shown in Figure 7c, in the state of eyes open, the signal amplitude was larger, and the blink waveform was obvious. In the state of eyes closed, the signal amplitude was smaller. In the human brain wave, alpha wave frequency ranges from 8 to 13 Hz. Alpha wave often appears in quietness and when a human closes eyes [36]. Figure 7d,e show the time spectra of the state in eyes open and eyes closed, respectively. In the state of eyes closed, the obvious peak in Figure 7b and the deeper red in Figure 7e, both at 10 Hz, represented higher energy of the alpha wave; which, is consistent with previous studies [37]. This EEG-collecting experiment proved the feasibility of the NEH self-adhesive electrode in collecting human bioelectrical signals.

### 2.8. Signal Stability of Time and Motion by the NEH Self-Adhesive Electrode

**Figure 8:**
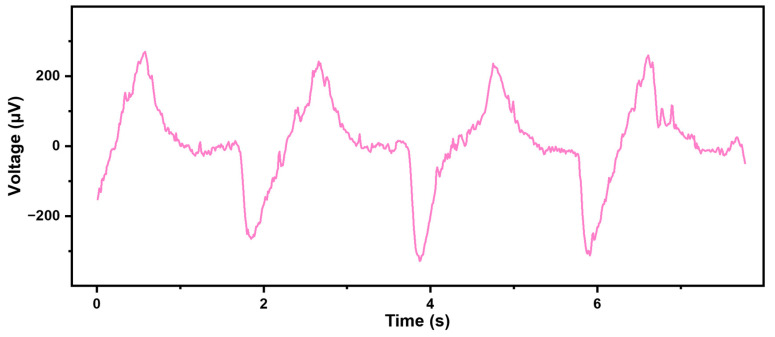
EEG signal recorded with NEH self-adhesive electrode after 3 days.

In order to prove the stability of the NEH self-adhesive electrode, we conducted experiments from two perspectives of time and motion (Figure 8). In terms of time stability, we kept the NEH self-adhesive electrode at room temperature (25 °C, 65% humidity) for 3 days, and then repeated the EEG collection experiment. The results were shown in Figure 8. The blink waveform barely changed compared with before (Figure 7c); which, was due to the stability of NEH. We also conducted experiments on the CEP to compare the difference between the NEH electrode and the CEP. After 3 days at 25 °C, 65% humidity, the CEP fell off from the subject when the non-woven had been removed before the test. We supplemented an experiment with a fresh CEP. Consequently, the fresh CEP without non-woven fell off again. The reason was that HCEP has poor mechanical strength. It was difficult to maintain the adhesion by merely relying on their low viscosity.

**Figure 9:**
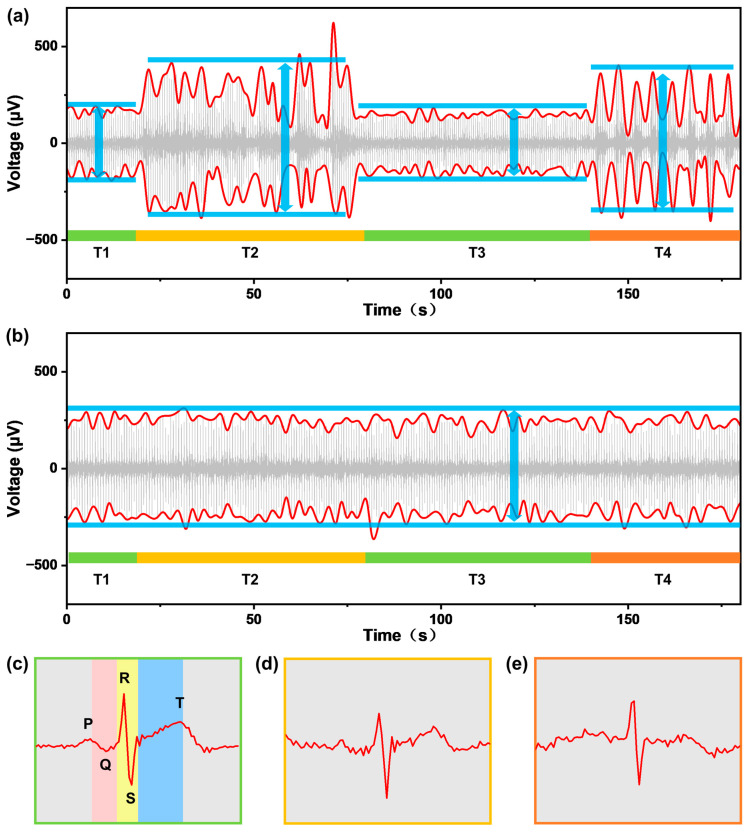
(**a**) ECG measurement with the CEP attached to the wrist. (**b**) ECG measurement with the NEH self-adhesive electrode attached to the wrist. ECG was obtained in three states: (**c**) static, (**d**) bending arm at 0.1 Hz, (**e**) bending arm at 0.2 Hz. The arrows represent peak-to-peak values of signals acquired at different actions. The capitalized letters represent different actions: static (T1), bending arm at 0.1 Hz (T2), static (T3), and bending arm at 0.2 Hz (T4).

Motions (such as movement, shake, and even vibration) commonly disturb the acquisition of biosignals to get distorted baselines and waves [38,39]. To verify the anti-interference ability of the NEH self-adhesive electrode, we collected the ECG signals under wrist motion disturbances (Figure 9). We placed the NEH self-adhesive electrode on the inside of the right wrist. GND electrode and Ref electrode (CEP) were placed on the back of the left wrist and the back of the left hand. The subject performed four actions as commanded: static (T1), bending arm at 0.1 Hz (T2), static (T3), and bending arm at 0.2 Hz (T4). The subject bent the right arm 90° in his direction, uniformly keeping his left arm still. Figure 9a shows the results of the CEP and Figure 9b shows the results of the NEH self-adhesive electrode. We can see that at stage switching, such as from T1 to T2, from T2 to T3, etc., CEP could not maintain a constant signal amplitude, as the red envelopes showed. In contrast, the NEH self-adhesive electrode always collected stable ECG signals. Besides, although in the static state, the amplitude of the signals collected by the NEH self-adhesive electrode was larger. The NEH self-adhesive electrode collected higher-quality signals than the CEP. As shown in Figure 9c, the NEH self-adhesive electrode sensitively acquired obvious P, Q, R, S, and T waves of ECG signals when static. The NEH self-adhesive electrode still obtained relatively complete ECG signals under motion interference in T2 and T4 (Figure 9d,e). Compared with HCEPs, the NEH had better mechanical strength to resist motion disturbances. High mechanical strength brought better toughness which can avoid slippage of hydrogel under motion disturbances. In summary, facing motion disturbances, mechanical strength is more important than viscosity for the electrode to stably acquire bioelectrical signals.

## 3. Conclusions

In this work, a new nanoclay-enhanced hydrogel (NEH) via simple one-pot synthesis has been explored for self-adhesive wearable electrophysiology sensing with high sensitivity and stability. Among existing hydrogels for the biological electrode, this NEH has outstanding mechanical performance (93 kPa of tensile strength and 1,326% of breaking elongation) and adhesion (14 kPa of adhesive force), owing to the design of a double-crosslinked constructure and the composite nanoclay, respectively. Furthermore, this NEH can still maintain good water-retaining property for the excellent long-term stability of signals, on account of the added glycerin. As a result, this hydrogel-based electrode can be applied for a wearable self-adhesive monitor to highly sensitively and stably acquire EEG/ECG electrophysiology signals of the human body over a long time. Last but not least, this hydrogel electrode can still acquire stable ECG signals under motion interference. This work provides a promising wearable self-adhesive hydrogel-based electrode for electrophysiology sensing; which will also inspire the development of new strategies to improve electrophysiological monitors.

## 4. Materials and Methods

### 4.1. Materials

Acrylamide (AM) was purchased from McLean Biochemical Technology Co., Ltd. (Shanghai, China). N,N′-Methylenebisacrylamide (BIS, 99%) was purchased from Aladdin Chemical Reagent Co., Ltd. (Shanghai, China). Deionized water, ammonium persulfate (APS), and tetramethylene diamine (TEMED) were purchased from Sigma Sigma-Aldrich Co., Ltd. (Shanghai, China). Sodium chloride (NaCl) was purchased from Jiangsu Qiangsheng Functional Chemical Co., Ltd. (Changshu, China) Glycerol was purchased from Sinopharm Chemical Reagent Co., Ltd. (Shanghai, China) Nanoclay (Laponite XLS) was purchased from Bike Chemical (Shanghai, China). The commercial electrode patch (CEP) was purchased from Shanghai Litu Medical Equipment Co., Ltd. (Shanghai, China).

### 4.2. Characterization

The microstructures of polyacrylamide hydrogels and NEH were studied by scanning electron microscopy (SEM, HITACHI S-4800, Tokyo, Japan). Before the examination, we froze and dried the hydrogel. The dried hydrogel was cut open to expose its internal structure, and its cross-section was observed by scanning electron microscopy. Infrared characterization was done by Fourier infrared spectrometer (PerkinElmer. Spectrum.100. Waltham, MA, USA).

### 4.3. Experimental Methods

#### 4.3.1. Preparation of the NEH

Firstly, AM (200 mg), NaCl (30 mg), nanoclay (5 mg), deoxidizing water (370 μL), and glycerin (450 μL) were added into a 1.5 mL centrifuge tube and stirred well to dissolve. Then, BIS (30 μL) was added for crosslinking and cooled the solution in the refrigerator (8 °C) for 30 min. Finally, TEMED (5 μL) as catalyst and APS (50 μL, 4%) as initiator were added. Then, the precursor solution was quickly poured into the prepared mold and placed the mold in a sealed bag for 2 h, 40 °C to form the NEH.

#### 4.3.2. Mechanical Performance

The mechanical properties of NEH were tested by XS (08) XG high modulus fiber strength instrument made by Shanghai Xusai Instrument Co., Ltd. (Shanghai, China). Dumbbell-type hydrogels (10 mm, 7 mm, 1 mm) were placed in the tensile jig at the speed of 10 mm·min^−1^ for the tensile property test. Each concentration sample was tested at least 5 times.

#### 4.3.3. Light Transmittance Property

Light transmittance property was tested by UV/VIS spectrophotometer (UV-5200PC) made by Shanghai Metash Instruments Co., Ltd. (Shanghai, China). The precursor solution was poured into the quartz cuvette. The quartz cuvette was put into the UV/VIS spectrophotometer until there was no air bubble in the precursor solution. The wavelength was 400–800 nm.

#### 4.3.4. Adhesive Performance

The adhesive performance of hydrogels was tested by lap shear. NEH (16 mm × 12 mm × 1 mm) was placed between two identical substrates of glass, silicone, stainless steel, pigskin, PVC, PP, PC, and PET (40 mm × 12 mm × 1 mm). A weight (500 g) was placed on the upper sheet for 3 min so that the hydrogel was in full contact with the sticky object. The computerized tensile testing machine loaded at a speed of 10 mm·min^−1^. Each concentration sample was measured at least five times, and in the obtained adhesion shear-displacement curve, the highest point in the curve represents the destruction of the lap shear specimen. At this point, the maximum force acts as the interface adhesion force.

#### 4.3.5. Preparation of the NEH Self-Adhesive Electrode

First, we used 3D printing technology to make the three-dimensional model as the mold. Then, we made the electrode substrate, including the stainless-steel electrode buckle, the Ag/AgCl electrode (ABS, surface with Ag/AgCl coating), non-woven fabric, and PET film. The non-woven fabric and PET film with a diameter of 16 mm were punched with a diameter of 3.5 mm in the center. The non-woven fabric was pasted on the PET film, and the Ag/AgCl electrode was combined with the electrode buckle through the non-woven fabric and PET film to form the electrode substrate. The substrate was placed in the mold, and the NEH precursor solution was added to be flushed with the upper surface of the mold. The mold was placed in a sealed bag and held for 2 h at 40 °C.

#### 4.3.6. Measurement for Conductivity

The ionic conductivity was measured using Multi-Channel Potentiostat/Galvanostat (PMC CHS08A). The ionic conductivity was tested using electrochemical impedance spectroscopy (EIS). To test the conductivity, we used a mold to make the hydrogel cylindrical for easy measurement. Its dimensions are 10 mm in diameter and 2 mm in height. We used two stainless steel sheets to sandwich the hydrogel in the middle. The test frequency was 10^0^–10^6^ Hz. The AC amplitude was 5 mV. Each sample was measured three times and three samples were tested to obtain an average.

#### 4.3.7. Measurement for Impedance

Impedance measurement included electrode pair impedance and skin–electrode impedance, obtained by using Multi-Channel Potentiostat/Galvanostat (PMC CHS08A) using electrochemical impedance spectroscopy (EIS). In electrode pair impedance measurement, two of the same electrodes were connected to be the electrode pair with hydrogels connected in opposite directions. Testing frequency ranged from 10^−1^ Hz to 10^4^ Hz, and AC amplitude was 5 mV. In the skin–electrode impedance test, the electrode under the test was the working electrode. The hydrogel of the CEP was removed to obtain a mold for NEH; in this way, we made the NEH electrode. The CEP was the counter electrode and the reference electrode. Test frequency ranged from 10^−1^ Hz to 10^4^ Hz, and AC amplitude was 5 mV. The test site was the forearm. The counter electrode, reference electrode, and working electrode are arranged in order. Soap was used to clean the oil and dirt on the skin before the test. We waited five minutes before testing and made sure that the water was completely dry. Each impedance test was measured at least three times.

## Data Availability

Not applicable.

## Supplementary Materials

The following supporting information can be downloaded at: https://www.mdpi.com/article/10.3390/gels9040323/s1, Figure S1. Adhesion shear-displacement curves at different substrates for (NEH-0.5%); Figure S2. Adhesion shear-displacement curves at glass substrate for NEH with different percentage content of nanoclay; Figure S3. Stress-stain curves for NEH with different percentage content of nanoclay; Figure S4. The conductivity test for NEH without NaCl and NEH with NaCl within 3 days; Figure S5. The gelation of hydrogel was activated by the nanoclay without BIS; Figure S6. Optical photos of precursor solution with nanoclay content at 0%, 0.2%, 0.5%, 0.8%, 1.0%, and 1.5%, respectively; Figure S7. Light transmittance results of precursor solution with different nanoclay content at 0%, 0.2%, 0.5%, 0.8%, 1.0%, and 1.5%, respectively.
