# Supplementary material for: A Nanoclay-Enhanced Hydrogel for Self-Adhesive Wearable Electrophysiology Electrodes with High Sensitivity and Stability
Source: Gels. 2023 Apr 11;9(4):323. doi: 10.3390/gels9040323 (PMC10137570; doi:10.3390/gels9040323)
Supplement: Supplementary file 1 [file gels-09-00323-s001.zip › gels-2297545-supplementary.pdf]

A Nanoclay-Enhanced Hydrogel for Self-Adhesive Wearable Electrophysiology Electrodes with High Sensitivity and Stability

Fushuai Wang [1,†], Lang Yang [2,†], Ye Sun [2], Yiming Cai [1], Xin Xu [3], Zhenzhong Liu [3,*], Qijie Liu [3], Hongliang Zhao [2,4], Chunxin Ma [2,3,4,*] and Jun Liu [1,3,*]

[1] Key Laboratory for Biomedical Engineering of Education Ministry, Department of Biomedical Engineering, Zhejiang University, Hangzhou 310027, China; 22160241@zju.edu.cn (F.W.); 22215040@zju.edu.cn (Y.C.)
[2] State Key Laboratory of Marine Resource Utilization in South China Sea, Hainan University, Haikou 570228, China; 21220856000067@hainanu.edu.cn (L.Y.); sunye@hainanu.edu.cn (Y.S.)
[3] Taizhou Key Laboratory of Medical Devices and Advanced Materials, Research Institute of Zhejiang University-Taizhou, Taizhou 318000, China; xuxinmark@163.com (X.X.); tanksman@163.com (Q.L.)
[4] Key Laboratory of Quality Safe Evaluation and Research of Degradable Material for State Market Regulation, Products Quality Supervision and Testing Institute of Hainan Province, Haikou 570203, China; hnxxzhao@126.com (H.Z.)
* Correspondence: zzliu@zju.edu.cn (Z.L.); machunxin@hainanu.edu.cn (C.M.); liujun@zju.edu.cn (J.L.); Tel.: +86-0576-88190681 (Z.L.); +86-0898-66292367 (C.M.); +86-0576-88190571 (J.L.)
† These authors contributed equally to this work.

This PDF file includes:

Supplementary Materials and Methods
Figure S1 to S7

Supporting Figures

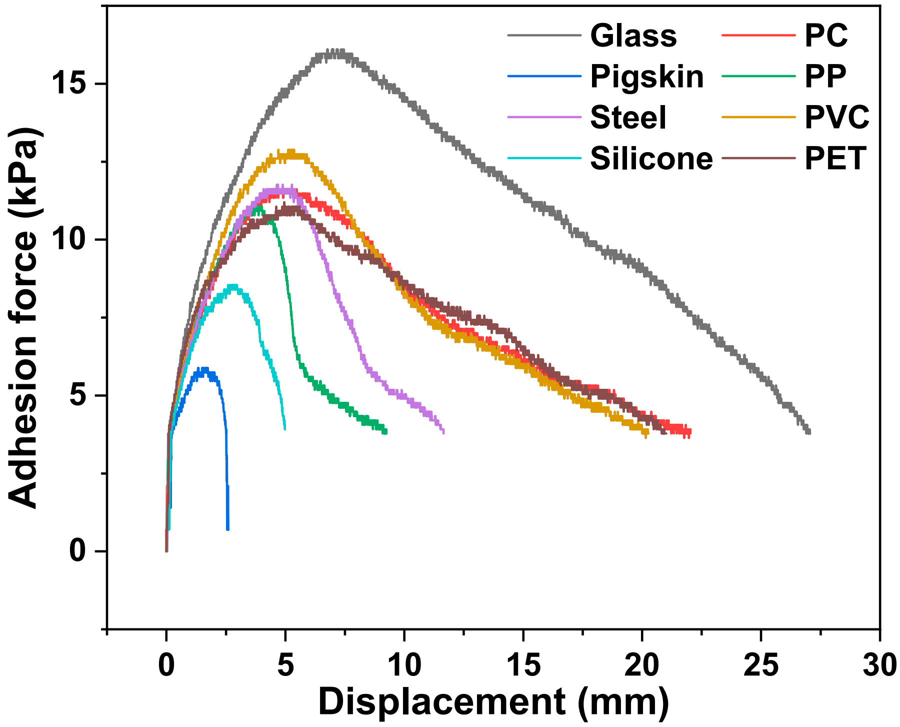

Figure S1. Adhesion shear-displacement curves at different substrates for（NEH-0.5%).

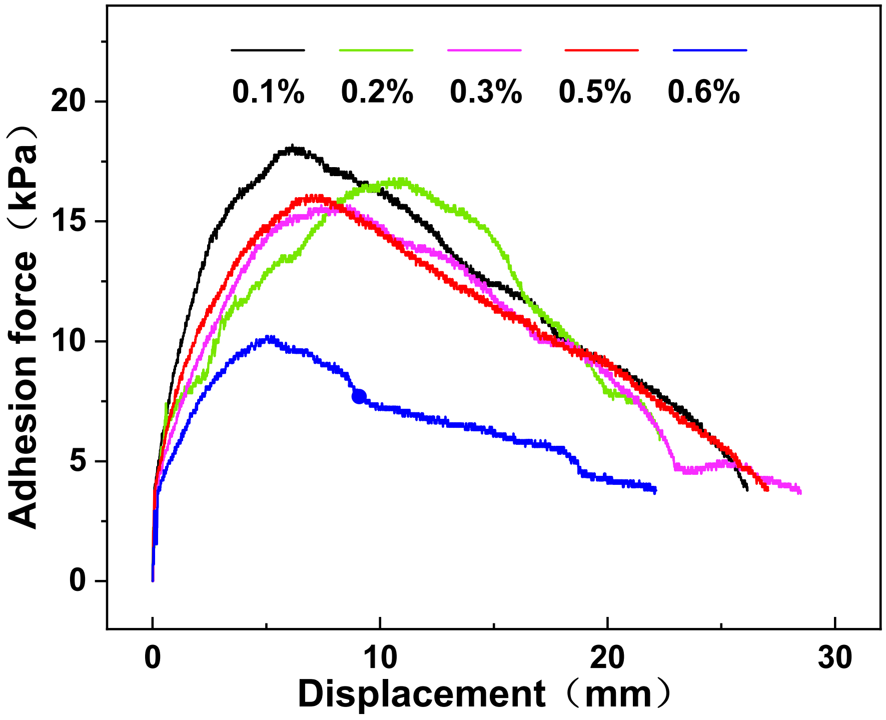

Figure S2. Adhesion shear-displacement curves at glass substrate for NEH with different percentage content of nanoclay.

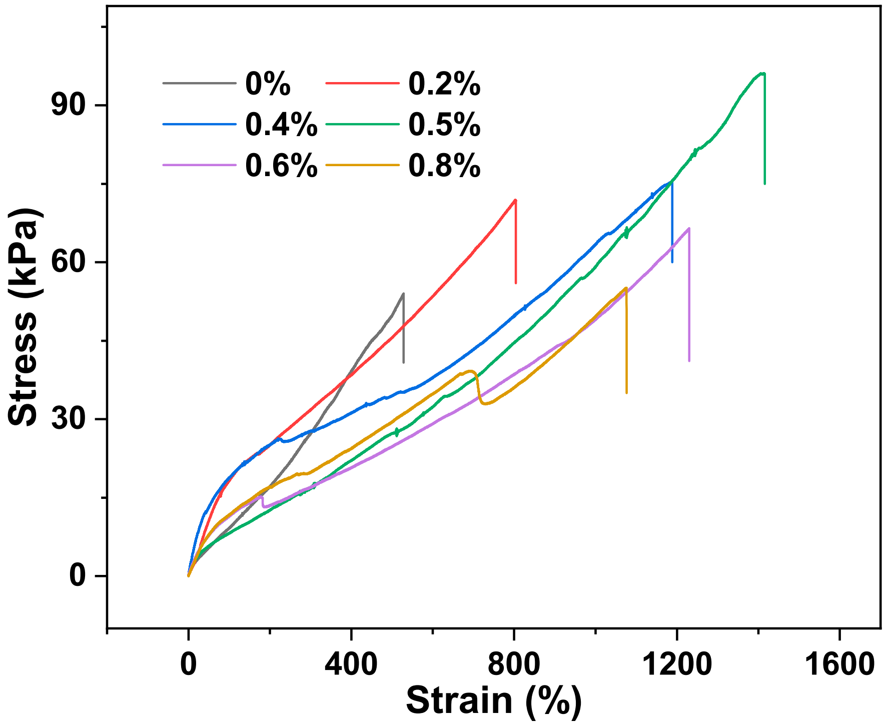

Figure S3. Stress-stain curves for NEH with different percentage content of nanoclay.

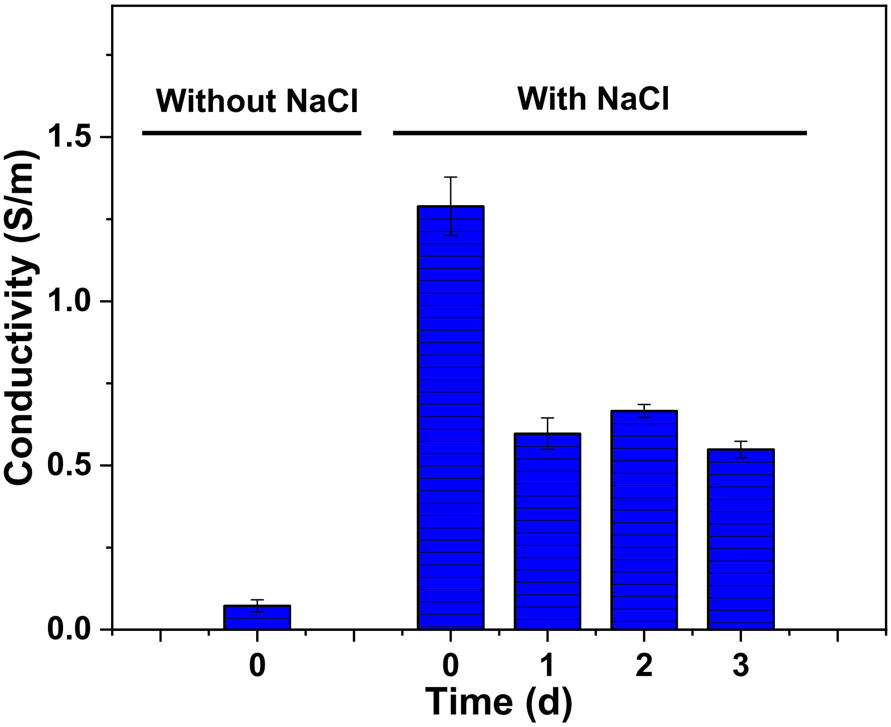

Figure S4. The conductivity test for NEH without NaCl and NEH with NaCl within 3 days.

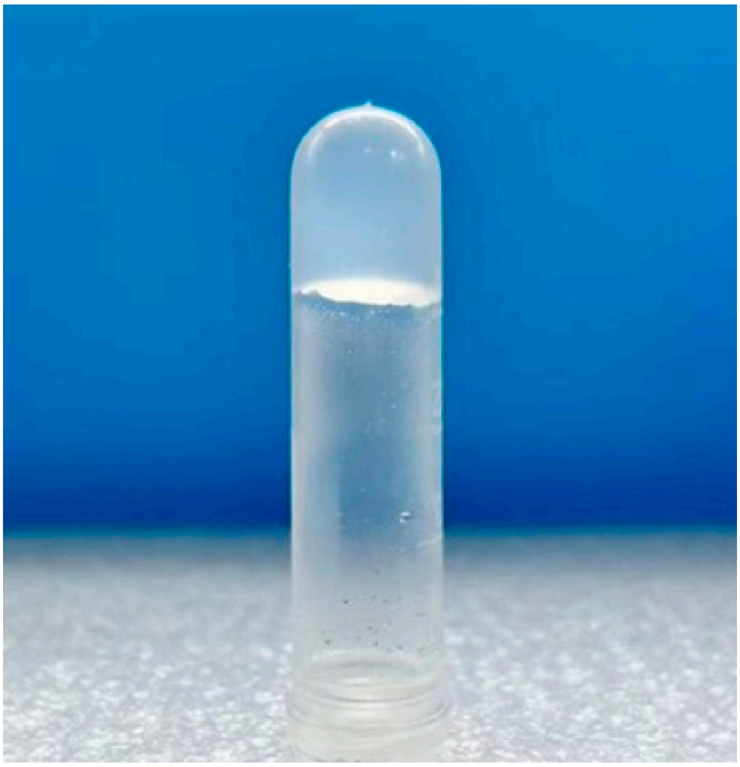

Figure S5. The gelation of hydrogel was activated by the nanoclay without BIS.

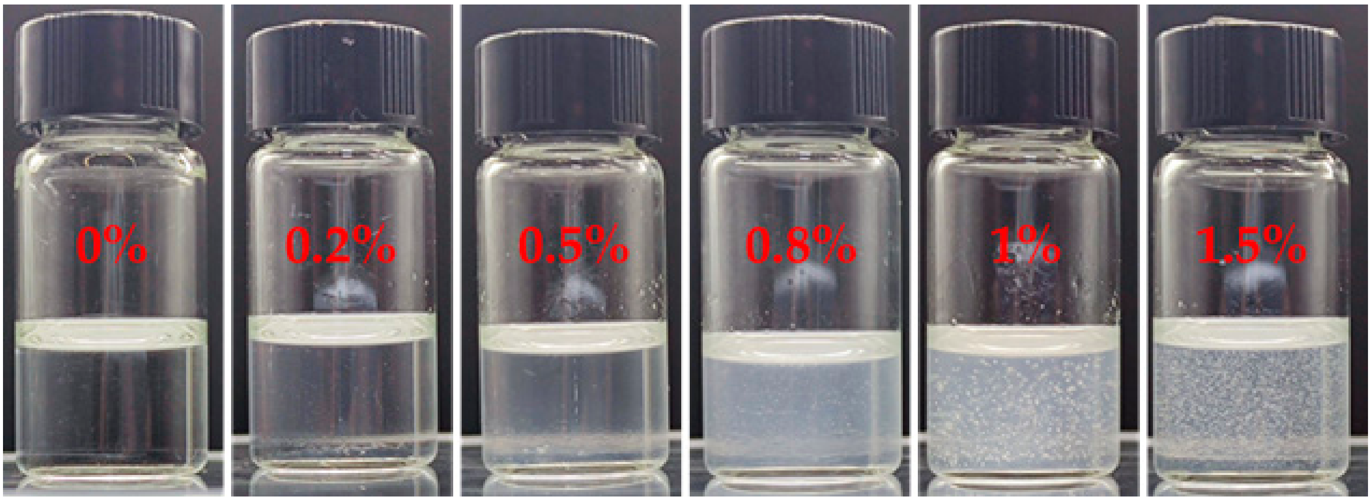

Figure S6. Optical photos of precursor solution with nanoclay content at 0%, 0.2%, 0.5%, 0.8%, 1.0%, and 1.5%, respectively.

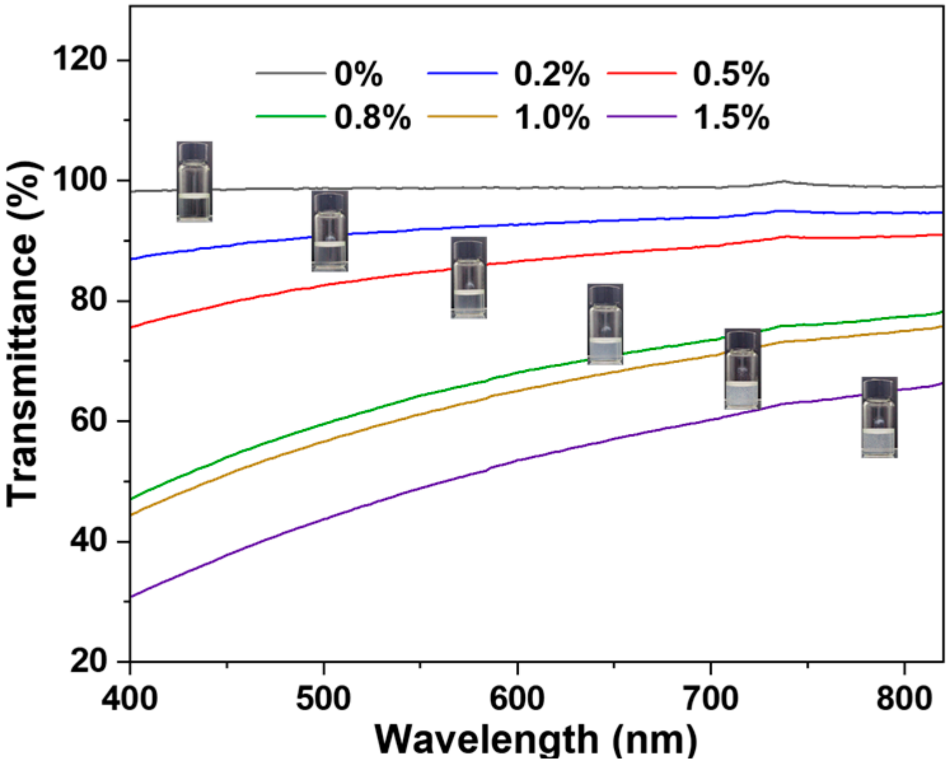

Figure S7. Light transmittance results of precursor solution with different nanoclay content at 0%, 0.2%, 0.5%, 0.8%, 1.0%, and 1.5%, respectively.
